# The Brittleness and Chemical Stability of Optimized Geopolymer Composites

**DOI:** 10.3390/ma10040396

**Published:** 2017-04-09

**Authors:** Michaela Steinerova, Lenka Matulova, Pavel Vermach, Jindrich Kotas

**Affiliations:** 1 Institute of Rock Structure and Mechanics, Academy of Sciences Czech Republic, v.v.i., Prague 18209, Czech Republic; matulova@irsm.cas.cz; 2 Department of Glass and Ceramics, Institute of Chemical Technology (ICT) in Prague, Prague 16628, Czech Republic; 3 Faculty of Civil Engineering, Czech Technical University in Prague (CTU), Prague 16636, Czech Republic; vermach.pavel@seznam.cz; 4 Department of Water Technology and Environmental Engineering, Institute of Chemical Technology (ICT) in Prague, Prague 16628, Czech Republic; jindrich.kotas@vscht.cz

**Keywords:** metakaolin, interfacial transition zone, compressive, flexural strength, elastic modulus, impact strength, acid leaching, porosity

## Abstract

Geopolymers are known as high strength and durable construction materials but have a brittle fracture. In practice, this results in a sudden collapse at ultimate load, without any chance of preventing the breakdown of parts or of withstanding the stress for some time. Glass fiber usage as a total anisotropic shape acting as a compact structure component should hinder the fracture mechanism. The optimized compositions in this study led to a significant reinforcement, especially in the case of flexural strength, but also in terms of the compressive strength and notch toughness. The positive and negative influence of the fibers on the complex composite properties provided chemical stability.

## 1. Introduction

The brittleness of geopolymers prevents them from being reliable components in horizontal constructions, such as a cross member for transversal beams and deck flue bricks [1]. Iron girders and steel concrete are utilized in practical situations, where their usage is often overdesigned. Currently, there is no material which could replace concrete with steel beams in cases of a lower load [2]. However, in spite of their brittleness, geopolymers are promising materials due to the variability of compositions and the possibility of combining other materials as reinforcement [3]. Fiber addition has been assessed several times with the publication of the mechanical properties, but no paper was found to have investigated reinforcement under the influence of different proportions of the composite components, while varying the addition of the glass-fiber reinforcement.

Paper [4] found that reinforcement by short glass fibers does not have any special effect on the increase in the compressive strength results, although it has a significant effect on the increasing flexural strength. The microstructure cracks are the focus of the investigation of the fracture mechanism [5], where the importance of the presence of glass fibers was shown on glass-fibers overcoming and fixing micro-cracks up to an overload of the fibers. The role of the interfacial transition zone (ITZ) was emphasized in the microstructure [6]. Whereas plastic fibers are used in Portland cement concrete, they are less suitable for the reinforcement of geopolymers as they do not bind tightly with geopolymers and water transportation takes place on the surface of the fibers during desiccation/polycondensation [7,8]. This is because the weakest part of the composite is the ITZ of the fibers, where the matrix has become porous. The resulting compressive and flexural strength do not correspond to the merit of the fibers’ addition. Better adhesion was found using polyvinyl alcohol (PVA) fibers [9]. Due to the related chemical composition of a geopolymer, glass fibers are more suitable than plastic fibers. Nevertheless, there is a thin alkali-resistant sizing [10] on the glass fibers, preventing mechanical strain during the fabric’s production. The sizing [11] is suspected to obstruct the binding of glass fibers to the geopolymer, which can lead to an increase of porosity. This is the reason why the glass fibers’ application was not as efficient as had been supposed.

**Figure 1:**
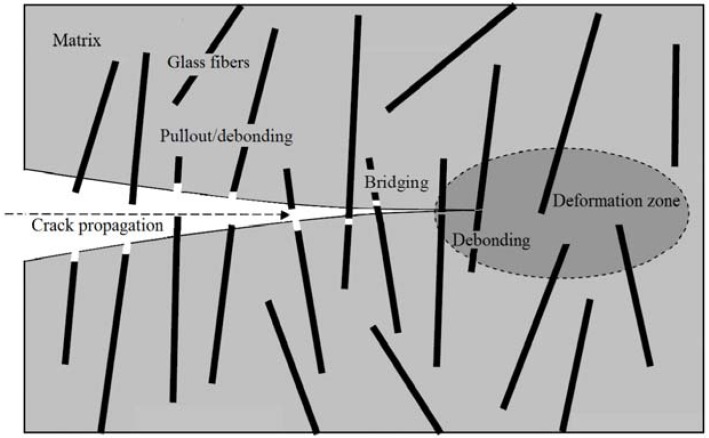
Fiber pullout and debonding in fiber reinforced geopolymer composites.

The contribution of short cut glass fibers to the properties of geopolymer quartz-sand mortars has been investigated in this paper. Mortars have recently been examined [12], during which the optimal composition was determined, and the grain size and the amount of aggregates were considered. The main goal of this work, however, is to identify the relationship between the geopolymer structure and the short cut glass fibers, to reveal the optimal ratio between the matrix, sand aggregate, and fibers, as well as to find an optimal technique for fabrication and to compare the effect of the aggregate-particle sizes. The fibers may have an influence on water transportation and involve the formation of a tight matrix microstructure in the ITZ by draining water during the polycondensation of the geopolymer gel. In fact, the glass fibers can bridge micro-cracks, which, more or less, are always parts of a geopolymer matrix and so influence the crack sorptivity and the products’ durability [13,14]. As has previously been found, the occurrence of fibers has an impact on decreasing the shrinkage [7]. This can eventually mean a lower number of micro-cracks and thus better mechanical properties. The cut fibers constitute extremely isotropic particles and the impact of the fibers on the brittleness of the geopolymer is examined. Figure 1 exemplifies the supposed behavior of the glass-fiber reinforcement in the geopolymer matrix, where the fibers are severed during the fracture while pulling them partly out of the matrix [15,16]. This grid is able to efficiently hinder the brittle fracture, in addition to improving the toughness of the composite structure, as is proven in our measurement. The combination of the varying ratios of the aggregate and fibers in the geopolymer was investigated by means of compressive strength, flexural strength, elastic modulus, and impact strength, using the Charpy method. The fiber addition suspected to be responsible for the increased capillarity was investigated in detail with the help of the resistance to acid waters and changes in the porous-structural parameters. The chemical stability and durability of reinforced composites was compared with a mortar without reinforcement. The intention was to demonstrate the acid resistance limited by certain structure parameters. We know that not all geopolymers are acid resistant, and at present, we are dealing with that issue in subsequent research focused on selected optimized compositions. The acid resistance in this paper is considered in order to compare the various mixtures with and without glass fibers.

## 2. Materials and Methods

### 2.1. Materials

As the initial materials, metakaolin, sodium silicate solution, and sodium hydroxide, were used to prepare the geopolymer binder. In this experiment, the molar ratio selected was Si/Al = 1.8, Na/Al = 1.0, and H_2_O/SiO_2_ = 0.7. Metakaolin was prepared from pulverized kaolin Sedlec Ia calcined at 750 °C for 6 h. The chemical composition of this kaolin is the following (wt %): SiO_2_ (47.3), Al_2_O_3_ (36.9), Fe_2_O_3_ (0.90), TiO_2_ (0.20), CaO (0.25), MgO (0.27), K_2_O (0.95), Na_2_O (0.02), and Loss by ignition (12.9). The particle size of metakaolin was 0.1–25 µm, with *D*50 = 15 µm.

The binder dispersion was stirred for 5 min under an intensive regime and successively under the vacuum removal of the air bubbles created by stirring. The quartz sand provided by Sklopisek Strelec, a.s. (Ujezd pod Troskami, Czech Republic), was added as the aggregate. The source was selected in three different types of sand, varying in grain-size distribution, and therefore, in the specific surface as a contact and reactive area (resulting into interfacial transition zone-ITZ), calculated from the grain-size measurement with the CILAS 940 and CILAS 2000 laser-dispersive granulometry equipment (Colas, Orleans, France). The sizes and specific surfaces are summarized below in Table 1.

Cut glass fibers of 4.5 mm in length (provided by Skolil-Kompozit s.r.o., Prague, Czech Republic) were added to the sand composite mixtures in two different portions, computed to the amount of geopolymer binder as a concentration of 5.0 and 2.5 wt % in relation to the mixture viscosity.

### 2.2. Sample Preparation

The usual ultimate addition of 5 wt % was due to the difficulty of the incorporation of the fibers into the paste, with the exception of the paste with coarse sand, which allowed an addition of 6.9 wt % through the diminished surface area of the coarse grains. Conversely, if the same amount of coarse sand was replaced with fine sand, the ultimate amount of the fibers was only 2.5 wt %, and the highest amount of 78 wt % of sand also allowed only 1.7 wt % of fibers, similar to the medium grain-sized mortar. For the thorough homogenization of the aggregates, the sand was mixed with glass fibers in a dry state. The prepared geopolymer binder was then added to the sand-fibers mix and kneaded well, in order to thoroughly shred the fiber bunches. Air bubbles and gaps were avoided by vibration. For a comparison of the fibers’ effect on the properties of geopolymers, three series of mixtures were manufactured according to Table 2, Table 3 and Table 4: composites with two different concentrations of fibers in the matrix and mortars without glass fiber addition. The addition of fibers without sand was not planned because of the sedimentation of the fibers and because the reinforcement was based on the various shapes of the aggregate, on the combination of spherical and isotropic particles. The prepared composites, M7-2.5* and M7-5*, exhibited a segregation of the glass fibers on the bottom of the specimens. Furthermore, it was ineffective to homogenize the reinforcement of mortars with less than 34 wt % of sand. Therefore, the process was continued with the addition of sand aggregate until the homogenization was reliable. Conversely, the mortar with 82 wt % of sand was not able to accept the glass fiber reinforcement.

The maximum glass fibers addition to the ultimate amount of fine and medium grain-sized sand was only possible at a value of 1.7 wt %. To simplify the figure captions, the results are shown in the columns assigned to 2.5 wt % in all of the diagrams, yet the discrepancy is only highlighted in the first graph legend. 

Three different dimensions of prism-shaped samples were manufactured into molds according to the given methods of the mechanical-property measurements described in Table 5. According to the standards used, three samples were produced from each mixture.

### 2.3. Measurement Methods

The compressive strength was measured by the standard CSN EN 196-1 [17] method on the EU40 testing machine provided by VEB Werkstoffprüfmachinen (Leipzig, German Democratic Republic). The flexural strength was carried out according to the same standard by three-point bending with the FPZ100 machine provided by VEG TIW Rauenstein Thüringer (Rauenstein, German Democratic Republic). The declarative measurement of the differences between the mix compositions was provided by the standard method on the specimens with a size of 40 mm × 40 mm × 160 mm, carried out by the accredited Experimental laboratory of Czech Technical University in Prague (FCE CTU, Prague, Czech Republic).

**Figure 2:**
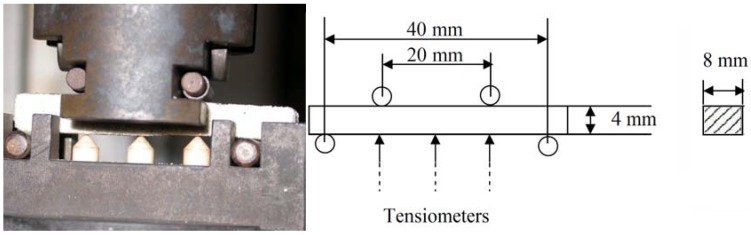
The dimensions of the samples for the measurement of the elastic modulus.

The elastic modulus was measured during four-point bending on the INSPEKT equipment by Hegewald & Peschke Meß- und Prüftechnik GmbH (Nossen, Germany), according to the intrinsic rules (Figure 2.). The sample size and shape were determined by the principal of the measurement of the four-point elastic modulus on this equipment. This methodology is based on the measurement of the glass-reinforced plastic of laminated shape, with a rule that the length of the sample must be at least twenty-times larger than the thickness. Under this ratio, the E-modulus depends on the ratio of length to thickness and the measurement is unstable.

The chemical stability of the composites was accomplished with the help of leaching in 0.2% HCl for one week at room temperature. The excess leaching solution was the same in all cases, denoting three samples of composites (10 g totally) in 50 mL of the acid solution. Leaching in acid water was undertaken to determine whether some of the composites from the prepared series endure the procedure and whether the structure records a measurable deterioration that would result in changes in the mechanical-strength and structural properties. In this experiment, the flexural strength and elastic modulus were measured after leaching and compared with the results measured on samples without leaching. A decrease was evaluated as structural decay. The measurement was carried out on 8 mm × 4 mm × 65 mm samples (according to Figure 2). In this case, the predominant orientation of fibers caused by shaping influenced the results of three-point bending if compared with the results reached for the 40 mm × 40 mm × 160 mm samples, but the measurement of the acid leaching impact conducted on the small samples is fully comparable and the results are reproducible. The immediate leaching of all samples occurred under the same conditions in the whole specimen volume and affected the small samples more intensively. Therefore, the experiment was relatively fast and the low deterioration had a huge influence on the changes of the structural properties and the bending strength, as well as on the E-modulus. The leaching waters left at the end of the test were compared in terms of their pH, using the Mettler-Toledo SevenCompact pH meter (Mettler-Toledo International Inc., Columbus, OH, USA).

**Figure 3:**
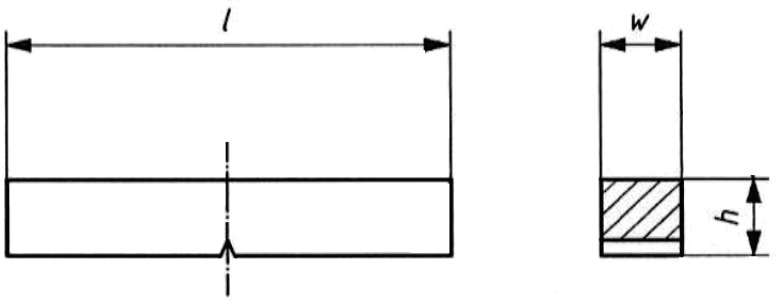
The dimensions of the samples for the measurement of impact strength by Charpy equipment; *l* = 55 mm; *w* = 10 mm; *h* = 10 mm.

Notch toughness was measured on Charpy equipment; for the sample sizes, see Figure 3.

### 2.4. Microscopy Observation

A scanning electron microscope (SEM) documented the microstructure by the Quanta 450 equipment from FEI company (Hillsboro, OR, USA). The heterogeneous matter was determined on polished cross-sections, which were vacuum coated with gold to prevent localized charging of the specimen and the consequent distortion of the electron beam, as geopolymers are poor conductors of electrons.

Optical microscopy of the composites determined the apparent texture of each composition with NIKON OPTIPHOT-100 microscopy (Nikon Corporation, Tokyo, Japan) in reflected light with a magnification of 50×. The images were shot by a JENOPTIK color digital camera (Jenoptik AG, Jena, Germany)and edited by the NIS-Elements AR system provided by Laboratory Imaging, s.r.o. (Prague, Czech Republic), for image analysis. As the representation in SEM produced less of an overview, the images were mostly collected by optical microscopy. The structures of the composites presented synoptic maps of all the composition objects, such as pores, aggregates, and microcracks in the contrast outlines.

### 2.5. Structure Determination

The structure was determined by high-pressure mercury porosimetry performed on a Pascal 240 device from POROTEC Vertrieb von wissenschaftlichen Geräten GmbH (Hofheim am Taunus, Germany). To determine the pore sizes, the ink-bottle computation model of pore shape was applied for adjoining the measured pressure of mercury to the appropriate size of the pores. The pore size distribution was recorded, to determine the influence of the reinforcement and aggregate addition on the structure parameters. The porosimetry was further recorded to detect deterioration after acid leaching.

### 2.6. Collection of FTIR Spectra

The changes in the structure of the sample were determined by Protégé 460 E.S.P. (Thermo Nicolet Corporation, Madison, WI, USA). Infrared Spectroscopy at an extension of 4000–400 cm^−1^, a resolution of 4 cm^−1^, and an average of 128 scans for the ATR mode with a diamond crystal.

## 3. Results and Discussion

### 3.1. Compressive Strength

**Figure 4:**
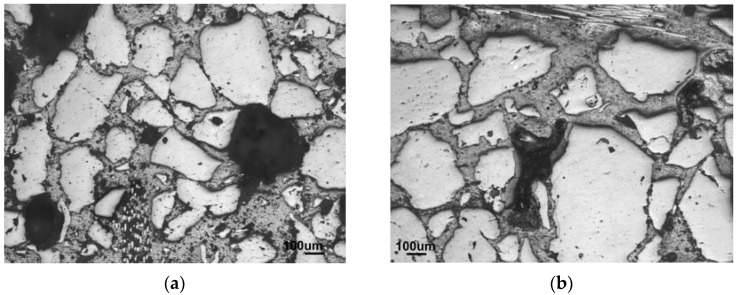
Comparison of the mostly densified structures of (**a**) fine-sand composite (F78-1.7) against the (**b**) less densified structure of medium-sand (M78-1.7) composite which was still able to reach 80 wt % of sand.

The reinforcement with 2.5 and 5 wt % of fibers, calculated as a concentration of the fibers in the matrix, was investigated in regard to the increasing amount of sand aggregates. The trend of the medium grain-sized sand series is compared to the alternatives with 70–78 wt % of fine and 70 wt % of coarse sand, instead of the medium one. Figure 4 compares the influence on the structure in the case of fine sand, where, although the portion of the matrix is the same in both structures, the particles are located closer together and the structure seems to be more densified in comparison to the larger grains of the medium sand.

**Figure 5:**
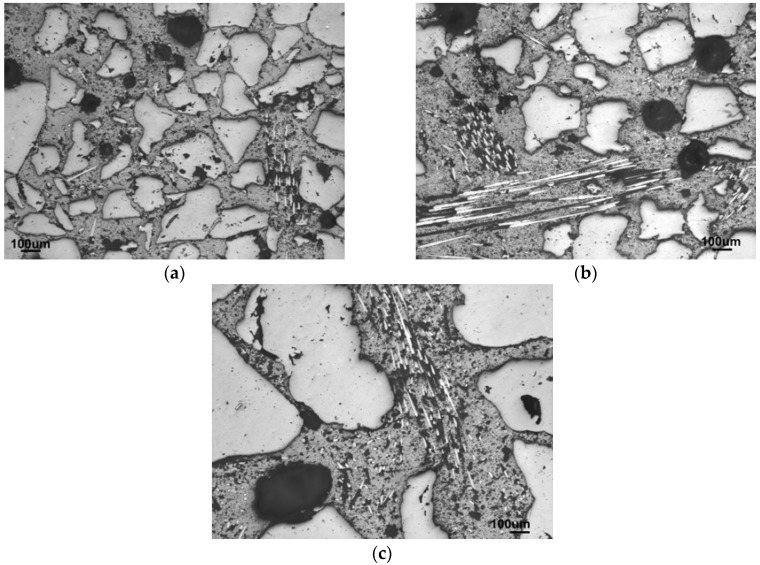
Various sand types of optimal amount with maximum possible fiber addition; (**a**) F70-2.5 fine sand; (**b**) M70-5 medium sand; (**c**) C70-6.9 coarse sand.

The addition of fibers which are efficient enough to improve the compressive strength requires an excess of the matrix to sufficiently coat the fiber surface. This also works improving the flexural strength and the lowering of brittleness. The coarse-sand mortar allowed an addition of 6.9 wt %, but the resulting strengths were weak due to the absence of smaller aggregate grains. The structures with the optimal aggregate amount of 70 wt % of differing grain sizes with the maximized addition of fibers, are shown in Figure 5. 

**Figure 6:**
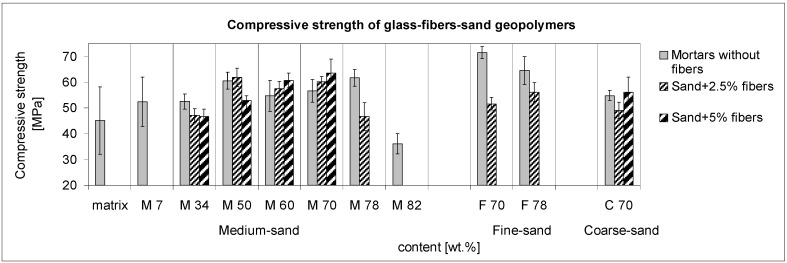
Contribution of glass fibers to the compressive strength of the geopolymer composites embodied by thin and bold hatching. The dependence of the sand aggregate increases with and without reinforcement of 2.5 and 5.0 wt % of fibers in the matrix measured on samples of 40 mm × 40 mm × 160 mm. The addition of the maximum glass fibers to the ultimate addition of fine and medium sand was only 1.7 wt %; 70 wt % of the coarse sand attained 6.9 wt % of glass fibers. The composites M7-2.5 and M7-5 were omitted for consideration because of the sedimentation of the glass fibers.

In the sense of the rising bulk density and shortening distance between particles, the compressive strength results are strongly dependent on the densification of the microstructure delimitated by the amount of sand and glass fibers, as shown in Figure 6. The fibers’ addition to the mortar of 70 wt % of sand (M70-5) reached the best compressive strength. The next composites and the mortar (M78) denote that the surface area of particles coated by the matrix is significant for shearing the stress under the ultimate pressure. The role of the interface area is emphasized by the mortars of fine sand (F70; F78), whereas the fine particles optimize the volume occupation of the structure. The fine-sand mortars were unsuccessfully reinforced by the fibers. It is clear here that only the balanced compositions optimize the mechanical properties. In the case of the fine-sand mortars, the wider scale of grain sizes enables a more densified order. Thus, the fine-sand mortar possesses the highest compressive strength without the reinforcement. The maximum addition of 5 wt % of fibers was more effective than the low addition of 2.5 wt % in all cases. The highest compressive strength, 63.5 MPa, was reached, denoting an improvement for 10 MPa, and an increase of 8.5%.

### 3.2. Flexural Strength

**Figure 7:**
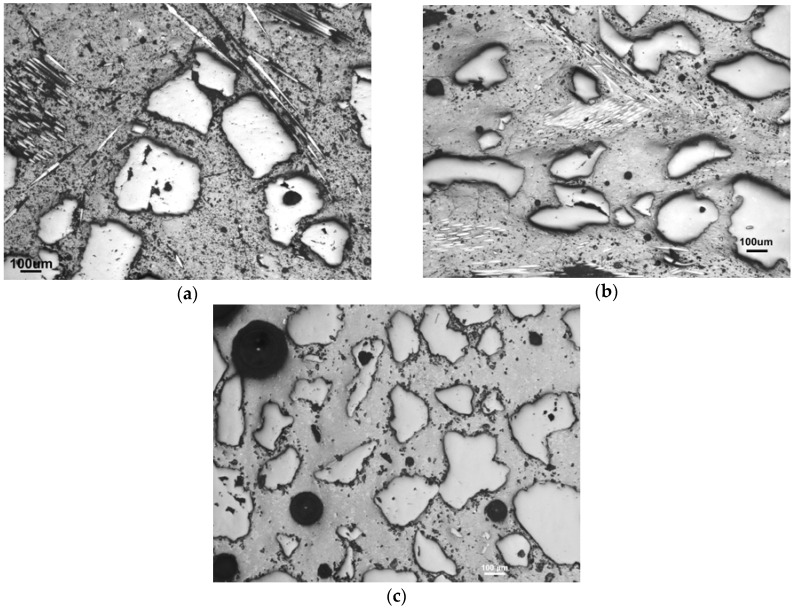
The porous structure of the fiber composites: (**a**) with a high amount of fibers in the matrix M34-5; (**b**) optimized composition M50-5; (**c**) mortar M50 without fibers.

Figure 7 shows the comparison of the porous structure caused by the hydrophobicity of the glass-fiber sizing. In the case of M34-5 (see Figure 7a), the amount of fibers was insufficient to balance the negative effect of porosity, whereas M50-5 (see Figure 7b) overcame the porosity by the amount of 5 wt % of fibers and thus presented the strongest mixture.

**Figure 8:**
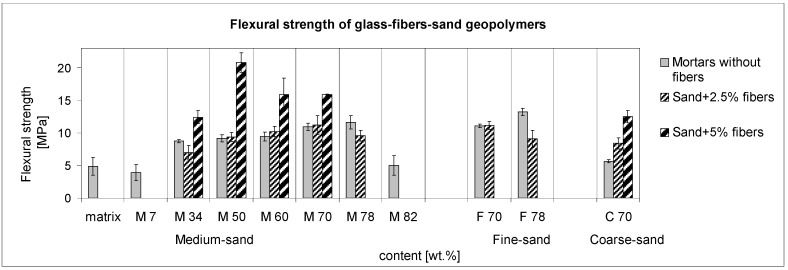
The flexural-strength trend of the sand composites reinforced with glass fibers. Measured samples size: 40 mm × 40 mm × 160 mm.

Figure 8 shows the results of the three-point bending strength. The mortars in the range M50-M78 maintained similar values of flexural strength up to the maximum value reached by M78. This indicates the influence of the aggregate surface area, which acts as an effective part of the structure by minimizing microcracks and bonding the quartz grains to the matrix by the functional ITZ. The addition of 5 wt % of fibers led to effective reinforcement, but at the expense of matrix consumption. M78 denoted a lack of matrix and a decrease in strength after the the addition of fibers. 

Fine-sand mortars reached the same flexural strength as the medium sand ones, but the reinforcement was less effective, due to the area already saturated by matrix without fibers. Coarse sand mortar (C70) was effectively reinforced by 6.9 wt % fibers, but only because the mortar possessed a low flexural strength. The fibers in C70-6.9 acted partly as reinforcement and partly as substitution for fine aggregate particles.

The addition of 5 wt % of fibers was mostly successful with 50 wt % of sand (M50-5), in contrast to the mortar M50, which, without fibers, reached only half of that value. Besides the leading role of the high amount of fibers, the requirement of the structure saturated by the matrix was still more effective than in the case of the compressive strength. Although in the case of M50 it was probable that the microcracks were more numerous than in the next mortars with 60–78 wt % of sand, fibers in M50-5 eliminated their negative effect. This implies that the high amount of aggregates allowing contact between the sand grains does not lead to flexural strength in the same way that it does to compressive strength, but the optimal surface of aggregates is significant. In addition, the connectivity of the aggregates with well coated fibers influenced the three-point bending with minimized microcracks. The addition of fibers of 2.5 wt % was less satisfactory than the addition of 5 wt % in all cases.

### 3.3. Brittle Fracture Decrease

**Figure 9:**
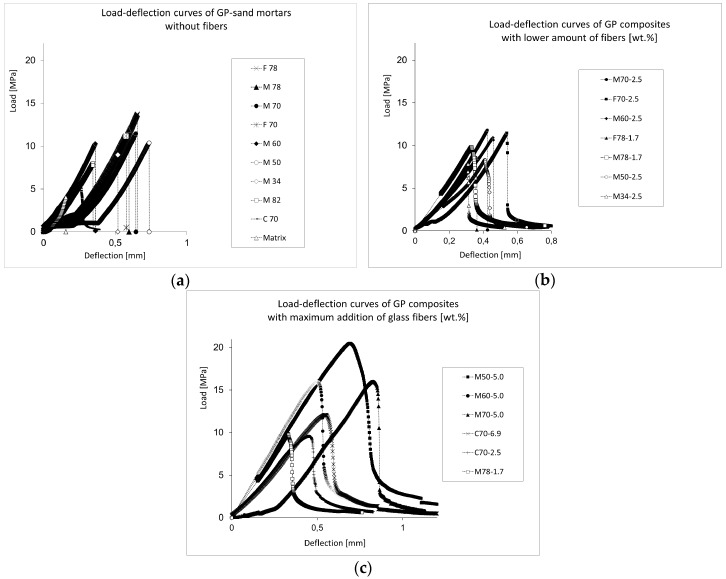
The comparison of load-deflection curves of geopolymer (GP) mortars (**a**) without fibers, (**b**) reinforced by 2.5 wt % of glass fibers and (**c**) with the maximum of reinforcement (5 wt %) with fibers (6.9 wt % in the case of the coarse sand).

The efficiency of the addition of fibers was confirmed by the load-deflection curves; see Figure 9. Due to the brittleness, all of the mortars’ load-deflection curves dropped sharply when the ultimate load was reached (Figure 9a). This implies an immediate break without the reinforcement of fibers. The crack propagation was only slightly suppressed in cases of the addition 2.5 wt % of fibers (Figure 9b). If composites contained 5 wt % of fibers (Figure 9c), the fracture extension was effectively suppressed, especially in a case of the composite M50-5, which also reached the highest flexural strength. Also, the coarse-sand composite C70-6.9 was effective in reinforcement, in contrast to the fine-sand mixture F70-2.5.

### 3.4. Impact Strength

**Figure 10:**
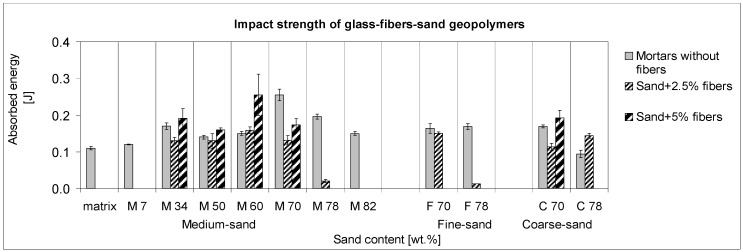
The trend of impact strength comparing mortars and glass-fiber reinforced composites.

The indicated impact strengths were quantified by the Charpy method as notch toughness, with the results shown in Figure 10. The trend of absorbed energy consumed for the fracture of samples after hammer impact suggested another fracture mechanism controlling the samples’ destruction than that which occurred during three-point bending. It is caused by the impact energy acting on the bonds as a shock. Whereas in the case of flexural strength the M50-5 showed the greatest toughness value, the composite with 10% more sand (M60-5) absorbed the most energy from the hammer impact. The resulting impact strength seems to be controlled by the intrinsic components’ brittleness besides the macropores involved in the fiber ITZ. Also, the lack of matrix led to less resistance, as the results with 70 and 78 wt % of sand show. This was in agreement with the much better resistance of highly reinforced coarse-sand composites C70-C78. As the fibers were brittle matter, their presence for impact-strength improvement seemed to be less effective than for flexural strength. Moreover, mortar without M70 fibers possessed the same impact strength as the M60-5composite. When considering the impact strength, fibers play a partial role as fine particles complementing the microstructure and partly as the buffer of microcracks. In cases of low additions (2.5 wt %), fibers actually decreased the impact strengths. Probably, the fibers affect the resulting impact strength through the ITZ macropores arose at the expense of the matrix mesopores.

### 3.5. Elastic Modulus

**Figure 11:**
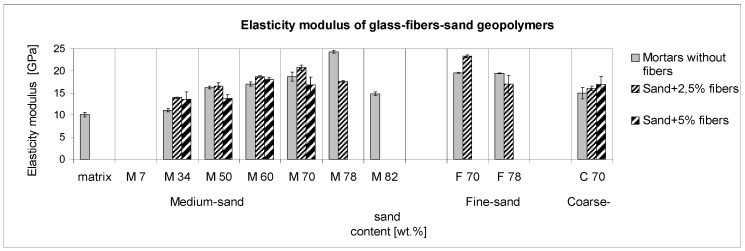
The trend of the reached elastic modulus depending on the composite composition.

The trend of the elastic modulus in terms of its dependence on the composite-parts ratio is provided in Figure 11. The effect of glass fiber addition to mortars seems to differ from the effects of the other mechanical properties mentioned above. The reason for this is that the measurement occurs without an overloading and breaking of the samples. As a reversible course, it depends on the structure properties, such as the elastic modulus of all the present components, their connectivity, and on a number of the connecting spots. The aggregate and fiber contacts with the sticking matrix are the control factor evidenced by the highest modulus detected at mortar M78; however, without fibers, and by the composite F70-1.7, which probably both contain a similar number of the sticking points regardless of the particle shapes. Concerning mortars with lower aggregates, the modulus is predominantly given by the components’ ratio, as the microcracks do not participate on any fracture. In addition, the elastic modulus was the only mechanical-property measure where, in all cases, the optimal addition of fibers was the lower one, that of 2.5 wt %. As it concerns composites with thoroughly saturated fibers, this behavior is probably caused by the low elastic modulus of the fiber glass. This is obvious from the fact that during the four-point bending measurement at the same forces, the reversible deformation of composites with 5 wt % of fibers was higher than in the cases of lower reinforcement. The tensometers recorded these differences in all cases, with the exception of the lowest aggregate amount. Here, the highest presence of fibers was evidenced by the proven highest deformation, which was similar in both cases of the addition of 2.5 and 5 wt %.

### 3.6. Acid Water Resistance

#### 3.6.1. Mechanical Properties after Weak Acid Leaching

**Figure 12:**
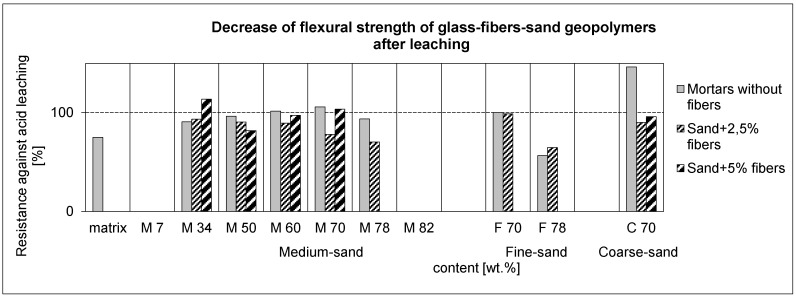
Resistance in acid water of 0.2% HCl as a decrease or increase of flexural strength. Comparison with and without fibers (composites and mortars), calculated as a ratio of the values after and before leaching.

**Figure 13:**
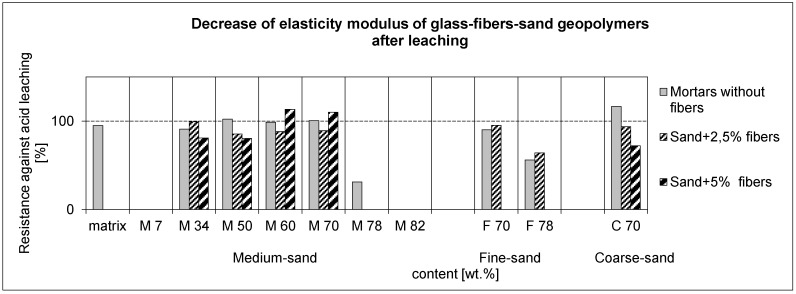
Resistance in acid water as a decrease or increase of the elastic modulus.

The measurement of stability against leaching in 0.2% HCl was carried out on small, slim samples with a size of 8 mm × 4 mm × 65 mm. The ability of glass-fiber reinforced geopolymers to withstand weak acid was demonstrated by the three-point flexural strength and elastic modulus; see Figure 12 and Figure 13, respectively. The results before and after leaching are compared by the decrease from the critical value of 100%, denoting the same value as before leaching. The mortars in the range M34-78 seemed to exhibit a better resistance without glass-fibers in an increasing trend of flexural strength, unless they were strongly reinforced by the maximum amount of fibers (M70-5; F70-1.7). Coarse-sand mortar displayed a high resistance, but it was the result with very low values of the flexural strength and elastic modulus before leaching. On the contrary, their high values (see the supplementary files) showed resistant composites M60-5, M70-5, and F70-1.7. The diminished cracks due to more aggregate tended to exhibit a better resistance.

#### 3.6.2. Structural Changes after Weak Acid Leaching

**Figure 14:**
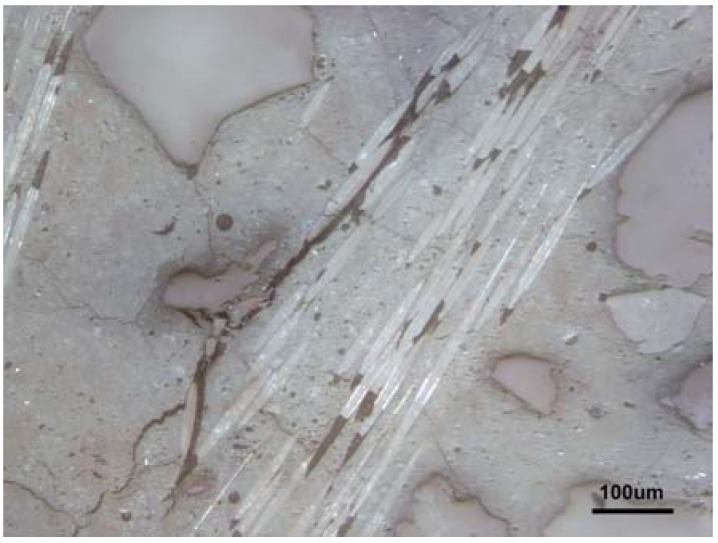
Matrix-fiber-aggregate connectivity against cracks in the composite structure. Sample M50-5.

Although the addition of glass fibers led to the decreased bulk porosity, partly through an addition of non-porous particles of fibers, in the range of macropores, the structure of the composites was more porous than the structure of mortars, as the results show. This was caused by the sizing on the fibers, which, due to its hydrophobicity, led to an increased presence of macropores, which was seen in the pictures of the microstructure. Besides the obvious presence of geopolymer microstructure parts, such as macropores and microcracks, their enlarged amount when conducting the fibers (Figure 14) contributed to the opened porosity and thus to the capillary sorptivity, which could ease the entrance of acid water.

**Figure 15:**
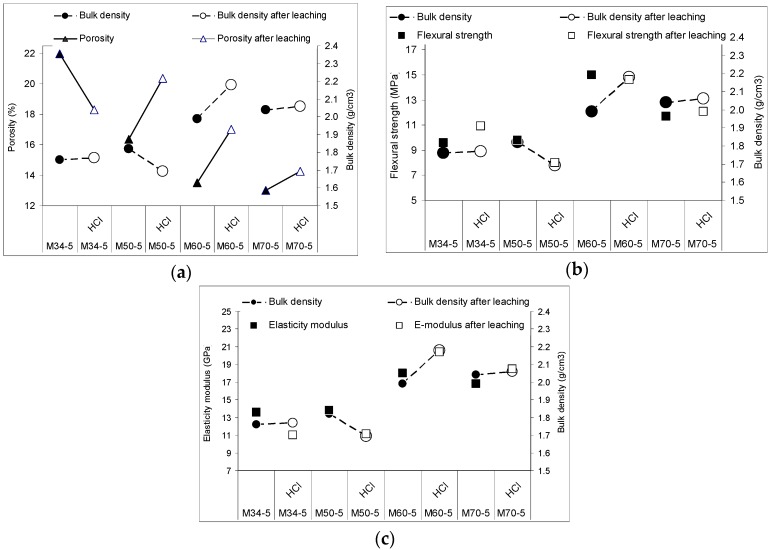
Correlation of bulk density before and after the acid leaching of highly reinforced composites with (**a**) raising porosity; (**b**) flexural strength and (**c**) elastic modulus.

To achieve resistance to acid waters, the balanced composition demands a sufficient amount of aggregate to minimize the microcracks and enough matrix to fill the volume among the aggregate grains (M50-M70). The disadvantage of the macropores arising from the fiber addition was overcome by the fiber maximum amount, which kept the M60-5 and M70-5 samples coherent through the leaching test. In the high amount of sand, the relative appearance of the added macropores was low (Figure 15a).

#### 3.6.3. Structural Changes after Weak Acid Leaching Compared to Mechanical Properties

It is obvious that the microstructure controlled the penetration of the leaching acid water and thus the mechanical properties after leaching. As Figure 15 shows, the opened structure embodied by the dashed line of bulk-density entered the expected fall after leaching (highlighted by the empty markers) in the composite M50-5. Here, the decrease of flexural strength and elastic modulus was in accord with the weakened structure. In any case, the other composites showed the trend that the reinforcement by fibers is effective, even after leaching, and helps the mechanical-properties performance, despite the structural deterioration by HCl. The reasons for this are the varying control plots affecting the mechanical properties within the changing composite components ratios, and thus, the structural parameters.

**Figure 16:**
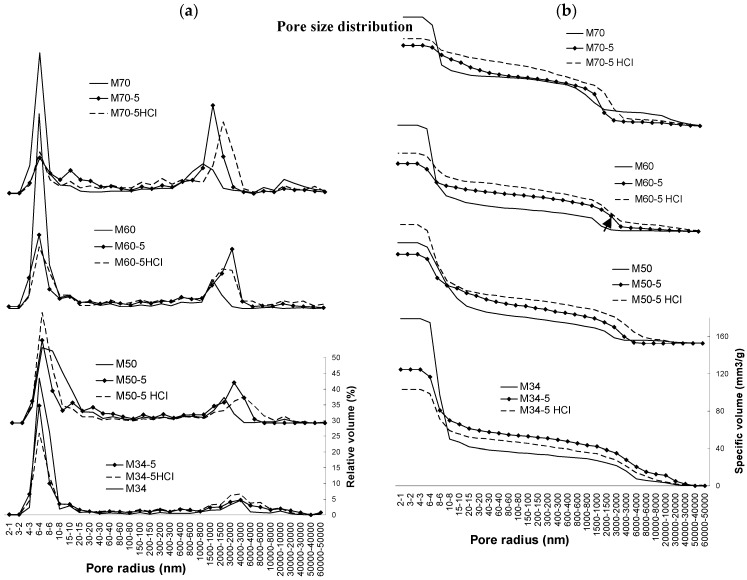
Pore-size distribution compared by (**a**) relative volume; (**b**) specific volume; the leaching noted the specific volume increase after leaching on all levels of pore sizes and at all composites, with the exception of M34-5, denoting its huge leaching.

Figure 15a points to a porosity decrease with an increased aggregate addition, conducted by bulk-density increase. Both parameters depicted reactions in the geopolymer matrix after one week of acid leaching in 0.2% HCl and subsequent drying. Although the fiber presence was supposed to raise the porosity and worsen the resistance in acid waters (Figure 12 and Figure 13), the maximum amount of 5 wt % of fibers in the matrix successfully achieved the aim of the process, and thus, the response of mechanical properties (Figure 15b,c). The trend of flexural strength reacted with the rise in the increasing sand amount, but the acid attacks, as the acid water leached the pores, caused an almost weakening effect. In the strongest sample M60-5, the acid-water resistance was acceptable, because the structure was probably not as leached as that with M50-5 with a higher porosity (Figure 15a). The elastic modulus (Figure 15c) even showed a strengthening and bulk density increase after leaching. This hints at chemical changes in the geopolymer composite, in the range of macropores and microcracks, which are effective at this ratio of aggregates and fibers (M60-5). Here, the porosity in Figure 15a shows that this value after leaching only returns to the value of M50-5 before leaching, while the strength remains high. This fact optimizes the composition of M60-5 for usage in an acid environment, for example, in mineral-water rich under the earth application such as in the mining industry. It is supposed that solvated amorphous SiO_2_ by acid leaching can be partly rinsed into the leaching water, and partly precipitated back into the structure. This could be a reason for the resulting bulk density (Figure 16a), as the value of M60-5 before leaching was 2.0 g/cm^3^ and 2.2 g/cm^3^ after leaching. Besides that, the continued polycondensation and solvated amorphous SiO_2_ probably led to blocked pores, or at least to their same size after leaching in a 3000–2000 nm range of macropores (bold arrow marked in Figure 16b), while reducing the bulk-density decrease due to leaching. In comparison to the M60-5 composition, in the sense of increasing bulk density, the composition M70-5 behaved similarly and its performance was optimized before and after the acid leaching (compare Figure 15 with Figure 9c). On the contrary, the performance of M50-5 shows sensitivity to leaching caused by the higher content of the matrix with relatively more microcracks, and after leaching the trend of mechanical properties worsened.

During leaching, the mass transport consisting of rinsing, solvating, and precipitating SiO_2_, led to minor changes in the microstructure parameters. FTIR spectra (see the Supplementary Materials Figure S1) indicated changes in the molecular reorganization of the matrix, showing a lower connectivity of SiO_4_ units (in a sense of decreasing the bridge oxygens number) as the wavenumber of the main peak T-O-T after leaching shifted from 980 to 1007 cm^−1^. A decrease of the wide peak at 3360–3380 cm^−1^, which can be ascribed to the occurrence of hydroxyl groups, points to the formation of the new bridging oxygens at the expense of the hydroxyl consumption. Carbonates were also detectable and their hydrates could also be responsible for the density increase. The only composition in which no density increase was detected after leaching, was M50-5, which (along with M34-5) noted the highest pH of the leaching water, representing the deterioration of matrix as the main part of the structure and can be seen in the trends displayed in Figure 12 and Figure 13. In this case, the porosity in the range of macropores seems to be higher than in the case of M34-5 and, thus, the more connected macropores are susceptible to leaching. It could explain the discrepancy in both of these porosity trends after leaching, while due to continuing polycondensation, the low aggregated M34-5 showed a porosity decrease opposed to the trend of M50-5 and others. In the case of M50-5, the more apparent the polycondensation was, the more the relative amount of mesopores was present (compare in Figure 16a), and it exhibited the highest peak in relation to all of the other composites in the 4–6 nm range of mesopores of. Nevertheless, the structural changes elucidated by the development of a specific pore volume versus the pore sizes (dotted lines in Figure 16b) showed in all cases, except M34-5, the porosity rising after leaching in the whole range of pore sizes.

In comparison to mortars (the full lines), the bulk pore-volume was diminished after the addition of glass fibers into all of the composites. The presence of the fibers, however, only increased the specific volume in the range of pores bigger than 8–10 nm in radius. It occurs at the expense of the smallest mesopores under 8 nm in radius, surrounding matrix clusters of which the porosity comprises the predominant part. See Figure 7c mentioned in the section on mechanical properties, where the structure is described by the differences between the mortars and composites with fibers.

### 3.7. Opacity and pH of the Leaching Waters

**Figure 17:**
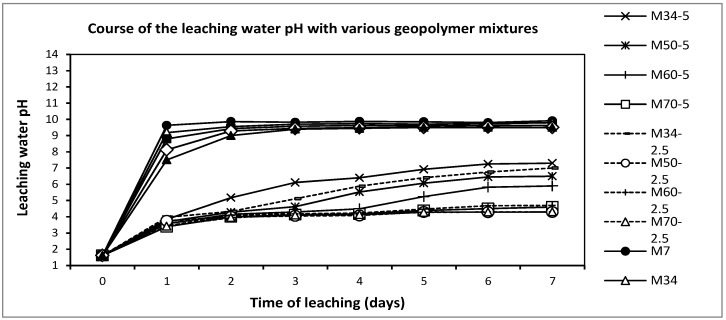
The recorded pH during one week of leaching in 0.2% HCl, comparing the differences between the geopolymer composites and mortars.

The final pH of the acid waters after one week of leaching was compared, keeping the ratio constant between the amount of the sample and leaching water with 0.2% HCl (Figure 17). Two classes of samples are shown; mortars after one day with a high pH and composites with a lower pH displayed for the whole week. Whereas mortars immediately released alkalis, composites were effectively reinforced by fibers bridging the microcracks, with a consequent delay of the acid water entrance. The fiber addition decreased the porosity, although fibers created new pores. In the case of the maximum addition of fibers (5 wt %), the new pores are actually responsible for the relatively high allowed entrance of acid water. Thus, this pH trend tends to neutralization more quickly than the pH of low reinforced composites (2.5 wt %), whose trends overlap at the lowest pH values. The observed opacity was caused by the acid precipitation of leached amorphous SiO_2_. It could be suggested that the opacity was highest in the case of a low amount of aggregate, despite the influence of the high pH. From these observations, it could be concluded that the trend showed the sensitivity of geopolymers to acid leaching to be strongly dependent on the presence of microcracks and connectivity of pores (capillary sorptivity). It could be claimed that geopolymer mortars with a high-aggregate ratio, with sand of fine and medium size grains, which implies a low amount of geopolymer high-pH matrix, are relatively resistant against matter release. On the other hand, too much aggregate in M82 led to the highest opacity due to the coarse pores. They denoted the highest sorptivity and leaching of matrix into the solution.

### 3.8. Microscopy after Leaching

**Figure 18:**
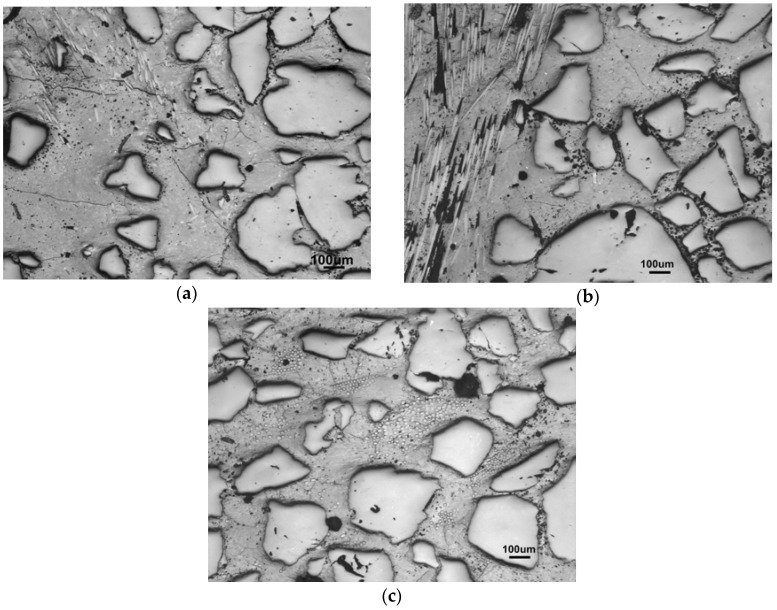
Comparison of composite M60-5 (**a**) before leaching; (**b**) M60-5 after leaching with fibers parallel to the cross section; (**c**) M60-5 after leaching with fibers upright to the cross section.

**Figure 19:**
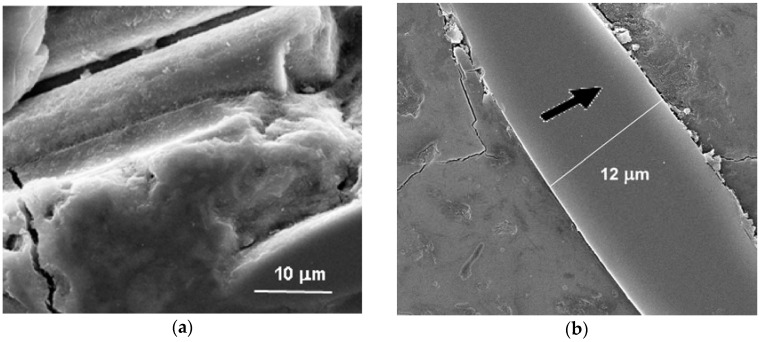
Scanning electron microscope (SEM) images of the encapsulation of fibers with a view of the fiber-matrix transition zone (**a**) of a fracture sample imagined in the secondary-electron modus; (**b**) of a polished section in the back-scattered electron modus showing the interfacial transition zone (ITZ) between the glass fiber and GP matrix being intact at the primary side of polishing and slightly deteriorated at the saw-runoff.

Macropores of a size of about 1–10 µm and more are visualized by optical microscopy as black dots in the relatively homogenous matrix of the composite M60-5 (Figure 18). The differences after leaching showed less structural changes, above all in a form of cracks and defects around glass fibers, caused by the process of polishing the cross sections, while the less optimized structures tended to have a defect occurrence during polishing, such as cracks opening and fibers breaking off. Therefore, only the best-performance composite M60-5 is imaged before and after leaching. However, in all cases, more defects occurred in the case of parallel laying fibers polishing; as shown in Figure 18b, the right-angle cross section completely avoided polishing defects. Generally, all the results were dependent on the optimal spread of glass fibers. The complex structure seems to be divided into a part rich in the aggregate and a part with only fibers, whereas the solitary fibers seldom enter between the aggregates. It is because the fibers stay in short bunches which obstruct shredding in the course of kneading the composite mass. Nevertheless, the fibers were satisfactorily coated by the geopolymer matrix and the bunches were well saturated, despite the low defibering (Figure 18c). The full-value contact between the glass fiber surface and matrix was pictured in great detail by SEM, as shown in Figure 19. The deterioration of the glass fiber edge was caused by polishing and is affected by the course of the cutting and polishing. The orientation of the sawing is highlighted by arrows.

## 4. Conclusions

The study presented the results of the mechanical properties and acid water resistance observations of geopolymer (GP) mortars reinforced with 2.5 and 5 wt % of glass fibers calculated as the concentration in the GP matrix in comparison with a series of mortars without fibers of the increasing amount of aggregate. The aggregate was quartz sand of varying grain sizes in an amount of 34 up to 82 wt % in the compositions.

As the results of mechanical properties showed, the structure after the addition of fibers was strengthened if the composition was optimally designed. The optimized addition was 5 wt % of glass fibers, the constant calculated as a part of the matrix, and the amount of the sand aggregates was optimal in the 50–70 wt % range.

The study showed the importance of the complex balanced ratios between compounds when a new additive is used. Beside that, the effect of the aggregate-particle size distribution was evaluated. The reinforcement was beneficial for the compressive strength and hugely so for the flexural strength, for effectively diminished brittleness confirmed by the load-deflection curves and also for the chemical stability in acid waters. Moreover, the composition, which satisfied the conditions, was only a part of all the possible mixtures. It was found that simply adding glass fibers into the GP mortar could worsen the composite mechanical properties and its acid water resistance. Another condition of the effective addition of glass fibers is the sufficient shredding of the fiber bunches, and when enough matrix thoroughly coated the surfaces of aggregate, the reinforcement fibers and the space between all the particles.

In terms of the compressive strength, the maximum of 64 MPa was reached with an amount of 70 wt % of the sand medium fractioned with matrix reinforced by 5 wt % of fiber. The best reinforcement reached an improvement of 8.5% in comparison to the mortar without fibers. However, the samples of finer sand (*D*50 = 0.28 mm) reached the absolute maximum of a compressive strength of 65 MPa containing 70 wt % of sand without fibers.

Focusing on flexural strength, the maximum of 21 MPa was reached. The composite of 5 wt % of fibers in the matrix contained 60 wt % of sand (medium fractioned grain-size *D*50 = 0.71 mm). The reinforcement oversized for 100% of the maximal flexural strength without fibers.

The presence of glass fibers is active in the fracture mechanism; above all, it is obvious in the strain-stress curves, in which the failure slowed down after the ultimate load. The best performance of fibers arose in the case of the fiber-reinforced composite of 50 wt % of sand medium fractioned. However, the maximum impact strength was reached at 60 wt % of the same sand with 5 wt % of fibers in matrix. Compared with the best value reached without glass fibers, the improvement of impact strength was small, of about 10%, reached at 70 wt % of sand. It implies that the courses of the stress-strain curve and impact strength were controlled by different plots acting under different destructive conditions. Whereas the three-point bending pulls the fibers out, the impact strength is more affected by the fiber-glass’ brittleness. 

The trend of the elastic modulus as a non-destructive method showed completely different relations. The highest stiffness pointed to the lower reinforcement by 2.5 wt % of fibers in the matrix. The reason for this lies in the intrinsic modulus of the fiber glass.

Although the glass fibers contributed to a significant porosity increase in the size range of over 10 nm in the radius of mesopores and above all in the range of macropores, the bulk porosity decreased with regard to the mortars. This is because macropores arose as the fiber ITZ at the expense of the GP matrix mesopores, which comprise the main part of the bulk porosity. The lowered porosity by fibers as a non-porous material is the minority.

It is apparent that the ITZ between the matrix, sand grains, and fibers is an important part of composites, affecting the mechanical properties and the acid water resistance. Through microscopy, the porous structure and the pH of the leaching waters revealed that fibers induced macropores, but bridged and reduced the microcracks, and thus improved the resistance of composites in acid waters. Despite the expected decomposition after leaching, as the macropores increased the porosity, the optimized fiber composite withstood the leaching, and even improved the mechanical properties and reduced open porosity. The likely reason for this might be the continuing polycondensation and amorphous SiO_2_ released by acid leaching, which could precipitate back into the interstitial space, in cracks, in mesopores, and probably partially blocked the macropores. This result can reveal a prospective use for geopolymers in the mining industry and for the fabrication of tunnel segments.

## Figures and Tables

**Table 1:** Sand aggregate properties.

Sand Type	Grain Size	Specific Surface
Medium	*D*50 = 0.41 mm	94 cm^2^/g
Fine	*D*50 = 0.28 mm	1518 cm^2^/g
Coarse	*D*50 = 0.93 mm	35 cm^2^/g

**Table 2:** Prepared samples—geopolymer mortars with quartz sand only.

**Sand Content (wt %)**	0	7	34	50	60	70	78	82
**Type of Sand (Medium)**	matrix	M7	M34	M50	M60	M70	M78	M82
**Type of Sand (Fine)**		-	-	-	-	F70	F78	-
**Type of Sand (Coarse)**		-	-	-	-	C70	-	-

**Table 3:** Prepared samples—geopolymer sand composites with low amounts of glass fibers.

**Sand Content (wt %)**	7	34	50	60	70	78
**Glass Fibers Content (wt %)**	2.5	2.5	2.5	2.5	2.5	1.7
**Type of Sand (Medium)**	M7-2.5 *	M34-2.5	M50-2.5	M60-2.5	M70-2.5	M78-1.7
**Type of Sand (Fine)**	-	-	-	-	F70-2.5	F78-1.7
**Type of Sand (Coarse)**	-	-	-	-	C70-2.5	-

* Note: glass fibers segregated on the bottom of the specimens.

**Table 4:** Prepared samples—geopolymer sand composites with maximal amount of glass fibers.

**Sand (wt %)**	7	34	50	60	70
**Glass Fibers (wt %)**	5.0	5.0	5.0	5.0	5.0
**Type of Sand (Medium)**	M7-5 *	M34-5	M50-5	M60-5	M70-5
**Type of Sand (Coarse)**	-	-	-	-	C70-6.9 **

* Note: glass fibers segregated on the bottom of the specimens; ** Maximum amount in the case of coarse sand.

**Table 5:** Dimensions of samples according to standards.

Measurement	Standard	Sample Dimensions
Compressive and Flexural Strength	CSN EN 196-1 [17]	40 mm × 40 mm × 160 mm
Flexural Strength, Elastic Modulus and Acid Water Resistance	Institute internal method	8 mm × 4 mm × 65 mm
Impact Strength by the Charpy Method	CSN ISO 148-1 [18]	10 mm × 10 mm × 55 mm

## Supplementary Materials

The following are available online at www.mdpi.com/1996-1944/10/4/396/s1. Figure S1: Fourier transform infrared (FTIR) spectra of geopolymer composite M60-5 with 5 wt % of glass fibers and 60 wt % of sand collected on the surface of the samples before and after leaching in acid water; Figure S2: Flexural strength of glass-fibers-sand geopolymers before (a) and after (b) leaching; Figure S3: Elasticity modulus of glass-fibers-sand geopolymers before (a) and after (b) leaching; Figure S4: (a) Decrease of flexural strength of glass-fibers-sand geopolymers afterleaching; (b) Decrease of elasticity modulus of glass-fibers-sand geopolymers after leaching.
