# Supplementary material for: The Brittleness and Chemical Stability of Optimized Geopolymer Composites
Source: Materials (Basel). 2017 Apr 9;10(4):396. doi: 10.3390/ma10040396 (PMC5506892; doi:10.3390/ma10040396)
Supplement: Supplementary file 1 [file materials-10-00396-s001.pdf]

Materials **2017**, *10*, *x*; doi:

Supplementary Materials: Brittleness and Chemical Stability of Optimized Geopolymer Composites

Michaela Steinerova, Lenka Matulova, Pavel Vermach and Jindrich Kotas

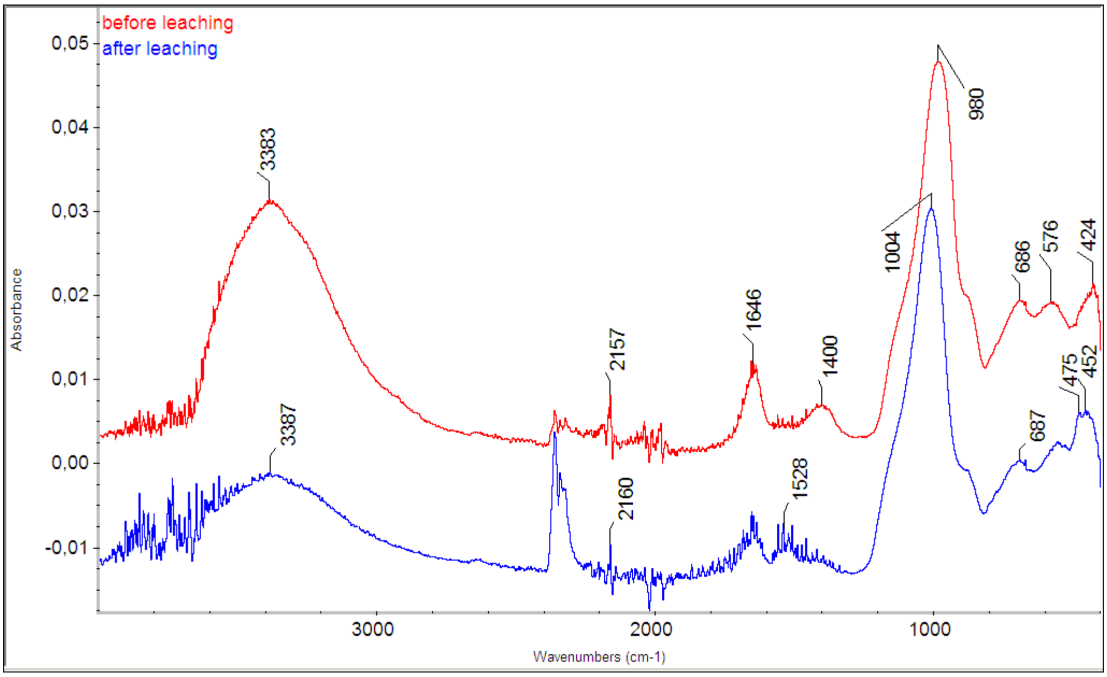

Figure S1. Fourier transform infrartd (FTIR) spectra of geopolymer composite M60-5 with 5 wt % of glass fibers and 60 wt % of sand collected on the surface of the samples before and after leaching in acid water.

(**a**)

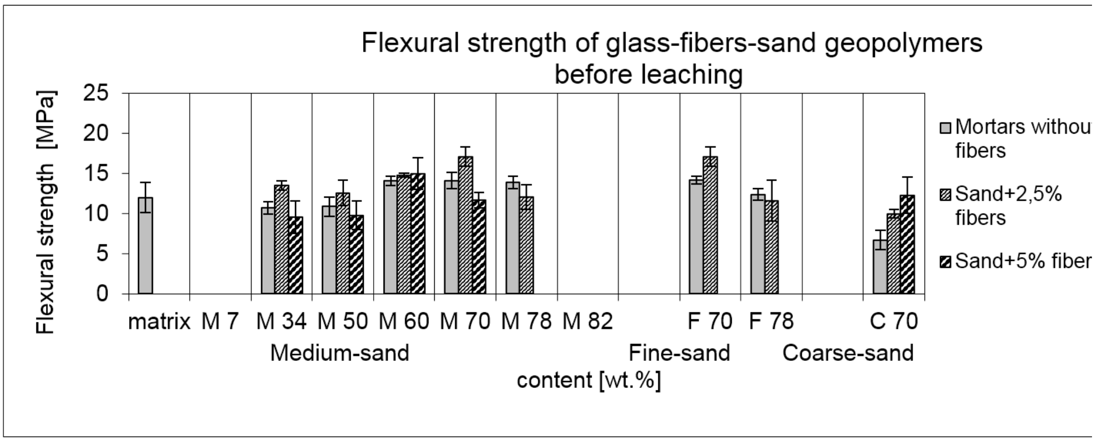

(**b**)

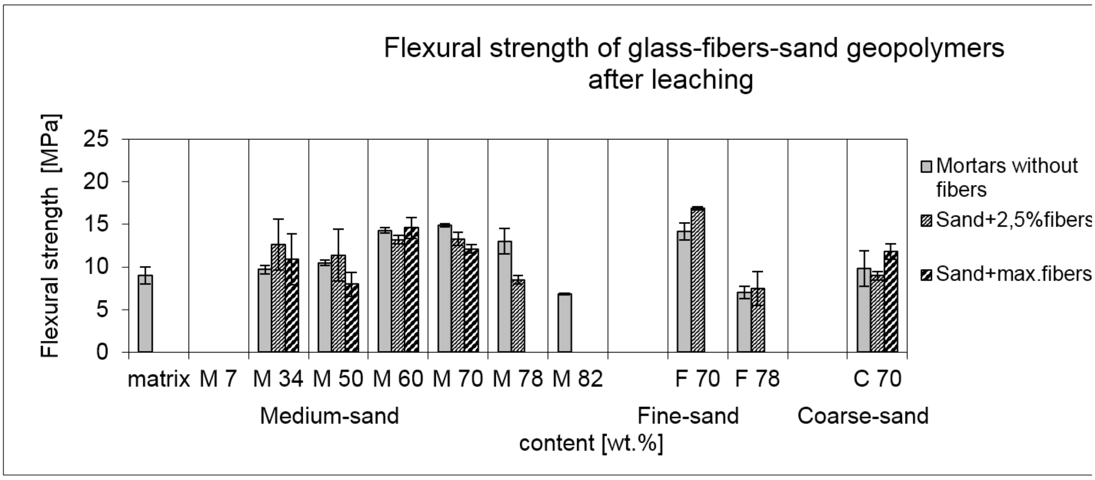

Figure S2. Flexural strength of glass-fibers-sand geopolymers before (**a**) and after (**b**) leaching.

(**a**)

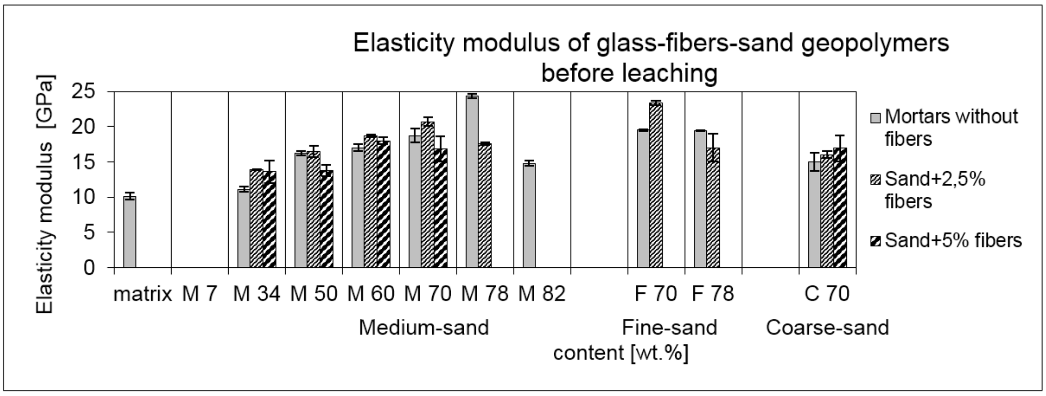

(**b**)

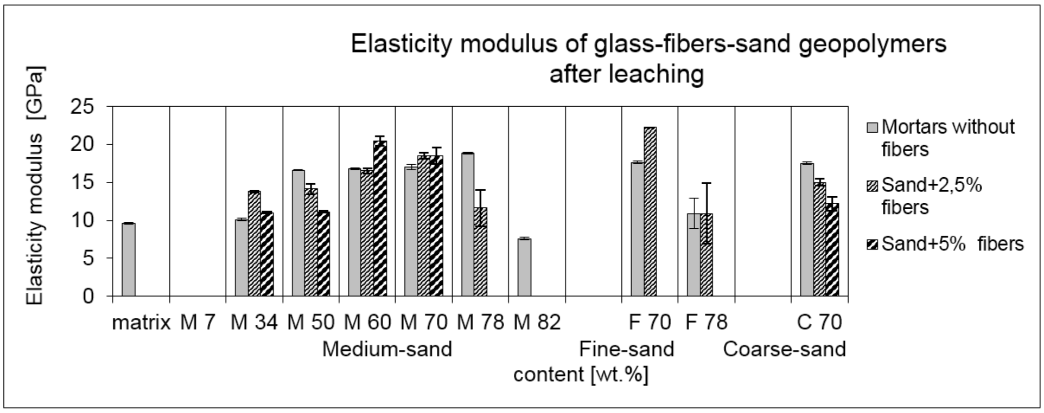

Figure S3. Elasticity modulus of glass-fibers-sand geopolymers before (**a**) and after (**b**) leaching.

(**a**)

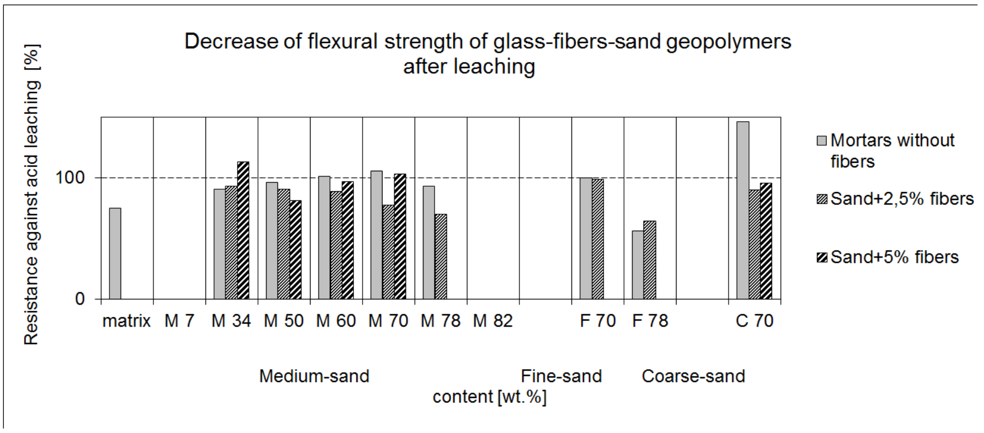

(**b**)

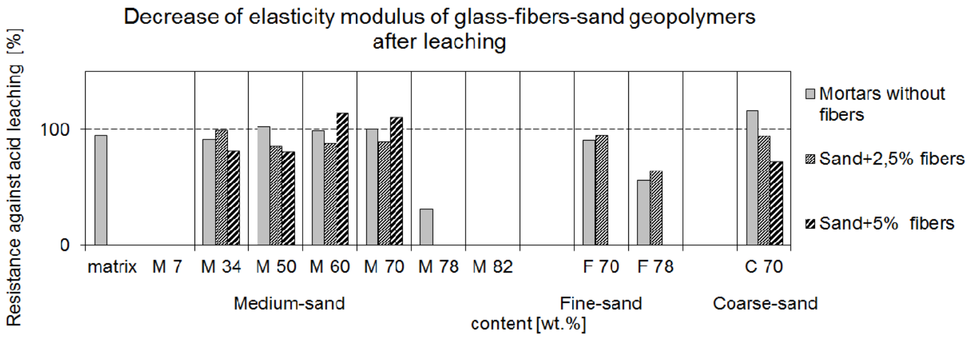

Figure S4. (**a**) Decrease of flexural strength of glass-fibers-sand geopolymers after leaching; (**b**) Decrease of elasticity modulus of glass-fibers-sand geopolymers after leaching.
